# Anatomy, Physiology, Hygiene, Etc.

**Published:** 1851-01

**Authors:** 


### ARTICLE VII.

*First Book on Anatomy, Physiology, and Hygiene, for Grammar Schools and Families.*

*A Treaties on Anatomy, Physiology, and Hygiene: designed for Colleges, Academies, and Families.* By CALVIN CUTTER, M. D., author of Anatomy, Physiology, and Hygiene, for Colleges, Academies and Families; second book on Anatomy, Physiology, and Hygiene, for Academies, Schools and Families; anatomical outline plates for schools, &c. Boston, Benjamin B. Mussey & Co. (From James Andrew, 42 West Randolph St., Chicago.)

Some one in commenting on the adage that, "A little learning is a dangerous thing," takes occasion to remark that no learning is still more dangerous, and we quite agree with him. At any rate it cannot injure boys and girls to study the fundamental truths of Anatomy, Physiology, and Hygiene. These volumes are well designed to fulfill this purpose, and we therefore cheerfully recommend them to the notice of Physicians, Teachers, and the community at large. M.

